# Hybridised production of technetium-99m and technetium-101 with fluorine-18 on a low-energy biomedical cyclotron

**DOI:** 10.1140/epjti/s40485-023-00089-2

**Published:** 2023-02-10

**Authors:** Erik V. Johnstone, Natalia Mayordomo, Edward J. Mausolf

**Affiliations:** 1 Innovative Fuel Solutions, LLC, Las Vegas, Nevada USA; 2 Institute of Resource Ecology, Helmholtz-Zentrum Dresden-Rossendorf, Dresden, Germany

**Keywords:** F-18, Tc-99m, Tc-101, Radiopharmaceuticals, Radiopharmacy

## Abstract

New modes of production and supply of short-lived radioisotopes using accelerators are becoming attractive alternatives to the use of nuclear reactors. In this study, the use of a compact accelerator neutron source (CANS) was implemented to explore the production of ^99m^Tc and ^101^Tc. Irradiations were performed with neutrons generated from a 16.5 MeV cyclotron utilising the ^18^O(*p*, *n*)^18^F reaction during routine ^18^F-fluorodeoxyglucose (FDG) production in a commercial radiopharmacy. Natural molybdenum targets in metal form were employed for the production of several Tc isotopes interest via (*n*, *γ*) reactions on ^98^Mo and ^100^Mo. The production of ^99m^Tc and ^101^Tc under these conditions is considered and discussed.

## Introduction

The capability of non-invasive, internal imaging, static or metabolic, of the various anatomical structures, processes, or the disease and detriment of these, has become the desideratum of diagnostics in the medical community. More than tens of millions of positron emission tomography (PET) and single-photon emission computed tomography (SPECT) procedures are administered every year in the United States of America (U.S.) alone. With the ever-growing demand for these procedures, the pursuit of innovative technologies and processes has become an incessant enterprise for establishing techniques and systems that are more work-efficient, cost-effective, and safety-conscious.

Technetium-99m (^99m^Tc, t$_{1/2} = 6.007$ h) has been widely used for radiodiagnostic purposes for decades, and it is still one of the most used radioisotopes worldwide constituting approximately 85% of all nuclear medicine procedures conducted. Tc-99m can be produced through various nuclear transmutation methods, but commercially speaking, it is generally derived from molybdenum-99 (^99^Mo, t$_{1/2} = 65.925$ h) via ^235^U targets [1]. However, the current commercial production and distribution of ^99m^Tc rely on a complex supply chain that has proven itself prone to disruptions in years past, which was most recently observed during the SARS-CoV-2 pandemic [2]. Ultimately, this leads to delays in the diagnoses of patients due to postponed imaging procedures as well as the loss of material and capital.

Compact accelerator neutron sources (CANS) have presented themselves in the last few decades as a potential alternative for the decentralised production and distribution of radioisotopes [3]. In this regard, CANS refer to an array of fundamentally different accelerator sources such as cyclotrons, radiofrequency quadrupole (RFQ) accelerators, linear accelerators (LINACs) coupled with a photoneutron converter, electrostatic accelerators, laser-driven sources, and neutron generators. With many of these systems, neutron fluxes upwards of ∼10^12^ n/s are achievable [4]. However, an under-utilised source of neutrons is those originating from (*p*, *n*) reactions during routine PET radioisotope production, such as for ^18^F-based radiopharmaceuticals [5].

For example, over the last few decades routine manufacturing of ^18^F-fluorodeoxyglucose (FDG), the most frequently implemented PET agent, has greatly evolved. From the benchtop synthesis of several doses at a time to hundreds of doses manufactured in a single production run with multiple runs daily, the evolution of ^18^F[FDG] has become the gold standard for in-house medical radioisotope production. At the present moment, there exists at least one of these facilities with a cyclotron in every single state in the U.S., where states with larger populations, and thus higher demand requirements, may have several. Therefore, the growth in ^18^F has signified a correlative increase in potentially available neutrons that are effectively not being utilised.

The use of complementary neutrons generated in a biomedical cyclotron employing the ^18^O(*p*, *n*)^18^F reaction has been proposed for radioisotope production [6], although its application for the production of ^99^Mo / ^99m^Tc or other Tc isotopes is hardly mentioned in the literature. In one study, ^99^Mo was implemented as a monitor for neutron flux in a Mo-containing multi-component flux wire measurement, although there was no targeted discussion for its production [7]. In another study, Link and Krohn report using neutrons generated during ^13^N production, i.e., ^16^O(*p*, *α*)^13^N, with an 11 MeV Siemens Eclipse at 30 *μ*A over a duration of 0.5 h for producing ^99^Mo / ^99m^Tc for teaching purposes. Although only small amounts were generated under these circumstances, the authors propose using (*p*, *n*) reactions and longer irradiations for higher output [8]. Considering the information provided in the literature pertaining to neutron production rates and energies, it was of interest to determine whether the simultaneous production ^99^Mo / ^99m^Tc and ^101^Mo / ^101^Tc with ^18^F in a biomedical cyclotron was feasible and if it could be a viable option for production and distribution of these medically relevant radioisotopes.

## Materials and methodology

**Figure 1:**
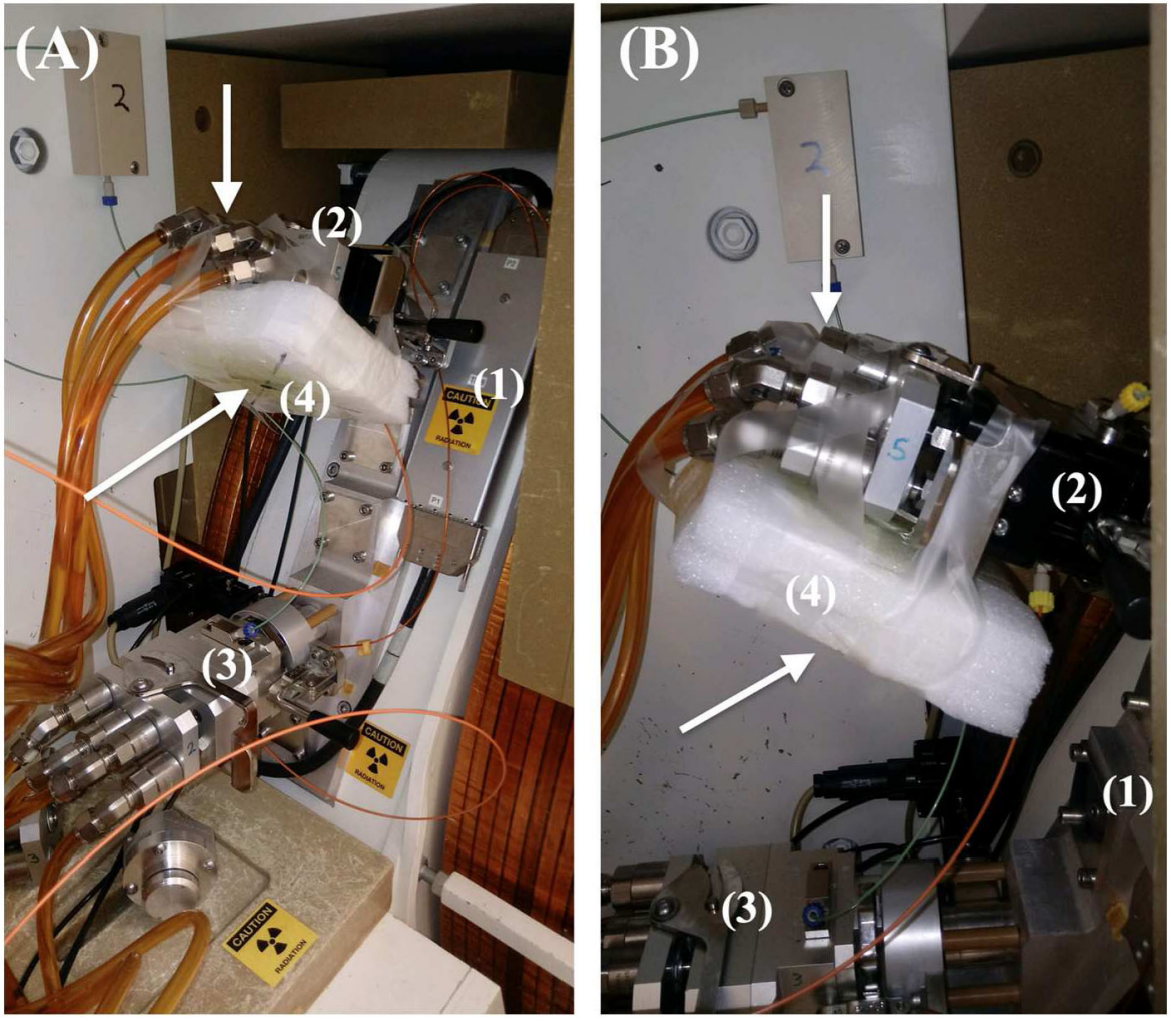
(**A**) Experimental setup for irradiation of Mo metal samples on a biomedical cyclotron (1) using high yield ^18^F targets from BTI Targetry. White arrows indicate the position of the samples to be irradiated on the primary ^18^F target (2) located in position 5, vertically located above a second ^18^F target in position 2 (3). The top arrow is pointing to the Mo metal block attached directly behind the target, and the bottom arrow is the location of the Mo metal foils behind a Styrofoam moderator (4). (**B**) Close-up view of the ^18^F target and Mo sample configurations from the perpendicular perspective relative to the incoming beam

Irradiations were performed with complementary neutrons generated on a biomedical cyclotron (Fig. 1) during routine ^18^F production with a ∼16.5 MeV proton beam operating between 75 *μ*A and 85 *μ*A. The ^18^F target material was ^18^O[H_2_O] with a purity of >98.0% ^18^O. High-yield ^18^F targets (BTI Targetry) were used [9]. The target bodies are made of Al 6061-T6 with a niobium (Nb) insert and implement a Havar alloy foil (0.04 mm) as a target window. Cooling of the target is performed using water and liquid transfer and pressurisation is used with high-purity helium (He) gas. The ^18^O[H_2_O] target volume is ∼3.5 mL.

Several varying geometries including cubes and foils of Mo metal were used for irradiations to determine the production of ^99^Mo. The Mo foil (10 cm × 10 cm × 0.01 cm) with ≥ 99.98% Mo was subdivided into 4-square pieces of dimension (5 cm × 5 cm × 0.01 cm) and approximate masses of ∼2.6–3.0 g. Mo target masses and dimensions are presented in Table 1. Foil samples were positioned underneath the ^18^F target in position 5 with a piece of Styrofoam moderator placed between the foils and the target. Mo cubes (volume ∼1 cm^3^, mass ∼10.3 g) with 99.95% Mo were used to determine production in thicker targets. Mo cubes were placed in small plastic bags and adhered to either the back of the ^18^F target directly (Cube 1) or behind the Styrofoam moderator (Cube 2) as shown in Fig. 1.

**Table 1:** Characteristics of Mo cube and foil samples used in irradiation experiments

Mo Sample	Mass (g)	Volume (cm^3^)	Surface Area (cm^2^)	Moderation
1—Cube	10.29075	1	1	No
2—Cube	10.26120	1	1	Yes
1—Foil	2.94533	∼0.25	∼25	Yes
2—Foil	2.59308	∼0.25	∼25	Yes
3—Foil	3.03616	∼0.25	∼25	Yes
4—Foil	2.52704	∼0.25	∼25	Yes

Irradiation durations and conditions were logged throughout the course of the workweek. The values generated, such as run time, ^18^F yield, and beam current, were used to determine relative neutron production rates and relative total neutron output in order to compare with measured values of activity generated in the Mo samples. Scheduled production of ^18^F in the facility involves at least two to three production runs in a typical working day. The cyclotron is equipped with two ^18^F targets located in positions 2 and 5 approximately ∼28 cm apart at the rear of the targets as shown in Fig. 1. For each working day, the two ^18^F targets were alternated for each corresponding run. Samples were positioned on the back of the ^18^F target in position 5 with the sample faces oriented perpendicularly to the direction of the incoming proton beam, as well as underneath the ^18^F target with the sample faces oriented parallel to the beam. Several of the samples underneath the ^18^F target were also positioned behind two pieces of a Styrofoam moderator to determine the effect of moderation. Although both sample sets were located in the closest proximity to the neutron flux generated on the target in position 5 (Fig. 1), it is acknowledged that the samples were also within the bounds of the irradiation field generated by the ^18^F target in position 2 during operation, albeit at a lower neutron flux and different energy regime.

Radioactivity in the Mo foils was quantitatively determined using a NaI-type gamma spectrometer, taking measurements at various time intervals after end-of-bombardment (EOB). The NaI spectrometer was calibrated with a ^137^Cs source from North American Scientific with an activity of 2118 Bq at the time of use. The detector efficiency was determined to be 10.814% for the ∼661 keV peak. It is acknowledged that at lower *γ* energies (E*γ*) better detector efficiencies are achievable, however, due to time and materials constraints of this study an efficiency plot was not established to determine this. The activities of ^99^Mo, ^99m^Tc, and ^101^Tc were determined using the gamma emissions at 181.1 keV (I$\gamma = 6.05$%), 140.5 keV (I$\gamma = 89.4$%), and 306.8 keV (I$\gamma = 89.4$%). The absolute activity (*A*) of ^99^Mo in several samples was calculated as a function of integrated counts under the corresponding peaks (*C*) and adjusted for background counts (*B*) at the region of interest (ROI), detector efficiency (*ε*), decay time after EOB until the measurement ($T_{d}$), decay during the measurement time ($T_{c}$), and the associated *γ*-ray emission probability ($I_{\gamma}$) and decay constant (*λ*) with each distinct isotope using Eq. (1) [10]. Furthermore, the reaction rate of ^99^Mo production in the irradiated samples was also calculated considering the irradiation duration ($T_{i}$) with Eq. (2) [10].

1 $$\begin{aligned} &A= \frac{\lambda (C-B)}{\varepsilon \mathrm{I}\gamma e^{-\lambda Td} (1- e^{-\lambda Tc} )}, \end{aligned}$$

2 $$\begin{aligned} &R_{Mo-99} = \frac{\lambda (C-B)}{\varepsilon \mathrm{I}\gamma ( 1- e^{-\lambda T_{i}} ) e^{-\lambda T_{d}} (1- e^{-\lambda T_{c}} )}. \end{aligned}$$

## Results and discussion

### Complementary neutron production on a low-energy cyclotron

At the fundamental level, the endoergic nuclear reaction of an impinging proton on ^18^O for the production of ^18^F can be represented by the equation:

3 $$\begin{aligned} ^{18} O + p \rightarrow ^{18} F + n,\qquad Q = -2.44\ \mathrm{MeV}. \end{aligned}$$

The reaction threshold energy is approximately 2.5 MeV with a reaction cross-section maximum of ∼550 mb occurring around 5 MeV. Although the ^18^O transmutation proceeds above and below the maximum cross-section energy, the construction of the target is devised so that the beam terminates within the water target, and it does so with a beam energy as close to the maximum cross-section. For a 16.5 MeV incoming proton beam, slowing of the beam, or beam degradation is performed within the Havar alloy window and controlled by its relative thickness. According to Eq. (3), the transmutation of ^18^O via proton capture is accompanied by the ejection of a neutron from the nucleus. Therefore, for every atom of ^18^F formed, a neutron is also generated. Thus, the degree of transmutation is dependent upon the number of impinging protons on the target, which can be calculated using Eq. (4).

Here, the elemental charge of a proton ($q_{\mathrm{proton}}$) is equal to $1.6 \times 10^{-19}$ Coulombs (*C*), and 1 microampere (*μA*) is equivalent to 10^−6^ C⋅s^−1^. The proton production rate ($R_{\mathrm{proton}}$) for a beam current ($I_{\mathrm{beam}}$) can be calculated as:

4 $$\begin{aligned} R_{\mathrm{proton}} \biggl( \frac{\mathrm{proton}}{\mathrm{sec}} \biggr) = I_{\mathrm{beam}} (\mu A)* 10^{-6} \biggl( \frac{C}{\mathrm{sec}} \biggr) * \frac{1}{q} \biggl( \frac{\mathrm{proton}}{C} \biggr). \end{aligned}$$

Under standard operating beam currents, $R_{\mathrm{proton}}$ values up to ∼1 × 10^14^ protons⋅s^−1^ are achievable. However, because of scattering events and interactions with the target body, window, and other components, the proton beam is not fully converted into ^18^F. One indicator of approximating beam conversion efficiency in the target is the saturation activity (*SA*), which is a function of ^18^F activity per unit of beam current, i.e., mCi⋅*μ*A^−1^. Using *SA* of the target and $I_{\mathrm{beam}}$ the number of ^18^F atoms produced per unit time can be determined, and thus the number of neutrons produced (R_*neutron*_) can be inferred as:

5 $$\begin{aligned} R_{\mathrm{neutron}} \biggl( \frac{\mathrm{neutrons}}{\mathrm{sec}} \biggr) = I_{\mathrm{beam}} *SA \biggl( \frac{mCi}{\mu A} \biggr) * 3.7*10^{6} \biggl( \frac{s^{-1}}{mCi} \biggr). \end{aligned}$$

For determining the thermal neutron flux ($\Phi _{\mathrm{neutron}}$, neutrons/cm^2^⋅sec) from *R*_neutron_ as shown in Eq. (5), Patterson’s formula [11] shown in Eq. (6) can be applied, where *K* (=1.25) is a constant and *I* is the surface area exposed to the neutron field.

6 $$\begin{aligned} \Phi _{\mathrm{neutron}} \biggl( \frac{\mathrm{neutrons}}{\mathrm{sec}* \mathrm{cm}^{2}} \biggr) =K \frac{R}{ I}. \end{aligned}$$

Thus, the higher the saturation activity, the higher the efficiency of beam conversion is and production of neutrons from the target. Likewise, the total activity generated per batch of ^18^F is equivalent to the total number of neutrons manifested. However, Carroll observed computationally with *ALICE9* that the correlation between neutron production and *SA* did not trend linearly when proton beam energies exceeded 12 MeV. This was attributed to other energetically accessible ^18^O(*p*, *x*) neutron-emitting channels that were not possible under 12 MeV [12]. Likewise, the neutrons resulting only from ^18^O transmutation do not completely account for the total production within the system. In fact, an array of (*p*, *n*) reactions occurs in the irradiation system, such as with nearby parts of the cyclotron or the ^18^O target including the Havar foil itself that interact with the stray proton beam. Accounting for the entirety of these possible interactions, the reported calculated flux produced from the production of ^18^F with a proton beam operating at 15 MeV and 75 *μ*A approaches 1.3 × 10^12^ n/s [13].

**Figure 2:**
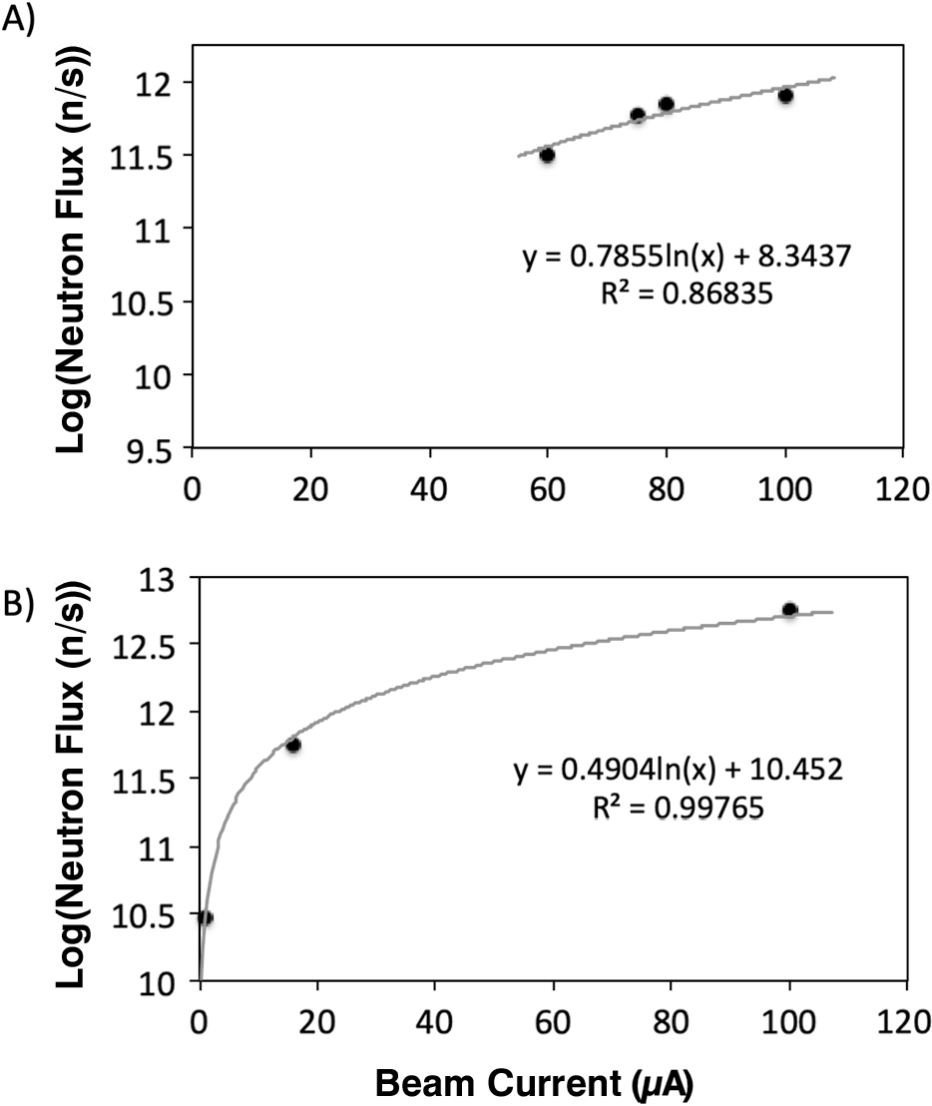
Reported neutron fluxes arising from the ${}^{18} O(p,n)^{18}$F transmutation reaction as a function of proton beam current for (**A**) 16.5 MeV and (**B**) 18.5 MeV energy cyclotrons [7, 12, 13, 15–18]

More accurate determinations of neutron fluxes produced in PET cyclotrons during ^18^F production have been conducted through experimental flux wire, neutron detection, and dosimetry measurements coupled with multi-system computational modelling, such as Monte Carlo, MCNP, FLUKA, etc. For example, Jeffries *et*. *al*. have reported the neutron flux generated from a GE PETtrace-800 employing BTI Targetry high yield ^18^F targets TS-1700 (80 *μ*A) and TS-1650P (72 *μ*A) [14]. Measurements were performed using a variety of activation flux wires with different neutron threshold energies along with comparative modelling via STAYS PNNL, MCNP6, and 3-Group programs. Results determined that the fast neutron flux density adjacent to the target was 1.8 × 10^9^ n/cm^2^⋅s to 3.0 × 10^9^ n/cm^2^⋅s with 1 MeV equivalent flux density from 2.4 × 10^9^ neutrons/cm^2^⋅s to 4.9 × 10^9^ n/cm^2^⋅s. Castillo analysed modelled data from a compilation of past studies concerning the neutron production on a 16.5 MeV proton beam on an ^18^O[H_2_O] target normalised to a beam current of 75 *μ*A [13]. A summary of the data shows that depending on the model employed and physical parameters integrated into the model, the $R_{\mathrm{neutron}}$ rate varied from 1.20 × 10^12^ n/s to 1.73 × 10^12^ n/s with neutron fluxes ranging from 2.18 × 10^8^ n/cm^2^⋅s to 1.45 × 10^8^ n/cm^2^⋅s. The determined neutron energy distribution spanned a broad energy range with maxima occurring in the epithermal to fast neutron regime, i.e., 0.01 to 5 MeV, as well as in the thermal region, ≤ 0.025 eV. Bosko [15] also reported on modelled neutron production rates and neutron energy distributions from a GE PETtrace-800. The $R_{\mathrm{neutron}}$ was determined for a 16.5 MeV proton beam on a thick ^18^O[H_2_O] target to be 3.21 × 10^11^ n/s assuming a 60 *μ*A beam current. The neutron energy distribution ranged from 1 MeV with a relative flux of ∼1.1 × 10^11^ n/s and extended to over 10 MeV with a gradual drop in flux to ∼1.1 × 10^8^ n/s; the majority of the flux focused in the neutron energy range from 1 MeV to 4 MeV. This $R_{\mathrm{neutron}}$ value is similar to the one reported by Horitsugi et al. from the GE Healthcare ALARA reports as 7.13 × 10^11^ n/s at 80 *μ*A during dual port irradiation [16, 17]. Figure 2 shows the correlation between proton beam current and the resulting neutron flux (n/s) for 16.5 and 18.5 MeV cyclotrons from reported values in the literature.

Another factor that should be considered is the neutron direction or angular distribution from the target. This is particularly important when considering the placement of a source to be irradiated, where neutron energy distribution and flux can be determined by sample position relative to the neutron source, i.e., ^18^O[H_2_O] target. For example, in a nuclear reactor scenario neutrons are emitted isotropically, whereas for accelerators this is not necessarily the case and neutron angular distribution is anisotropic. For accelerators, some of the energy from the primary incoming beam will impart some of its energy on the secondary particle emitted, which can lead to scattering as well as backscattering of the secondary particles. Generally, the higher the energy of the incoming proton, the higher the energy of the neutrons produced.

For the transmutation of ^18^O to ^18^F with a high-energy proton beam, the majority of the generated neutrons are emitted in the same direction as the incoming proton beam. As mentioned previously, scattering is observed further out from the source origin in the ^18^O target, where the neutron field becomes more diffuse, although still mostly directionally forward. It is also noted that a lesser, yet significant amount of neutrons are emitted in the opposite direction, or essentially backwards from the target into the incoming proton beam. Specifically, the GE Site Planning Guide states that when compared to the total flux of neutrons in the forward direction, those perpendicular to the incoming particle beam will be 30% less of this value, and those in the backwards direction will be at least 10× lower in magnitude [18].

**Figure 3:**
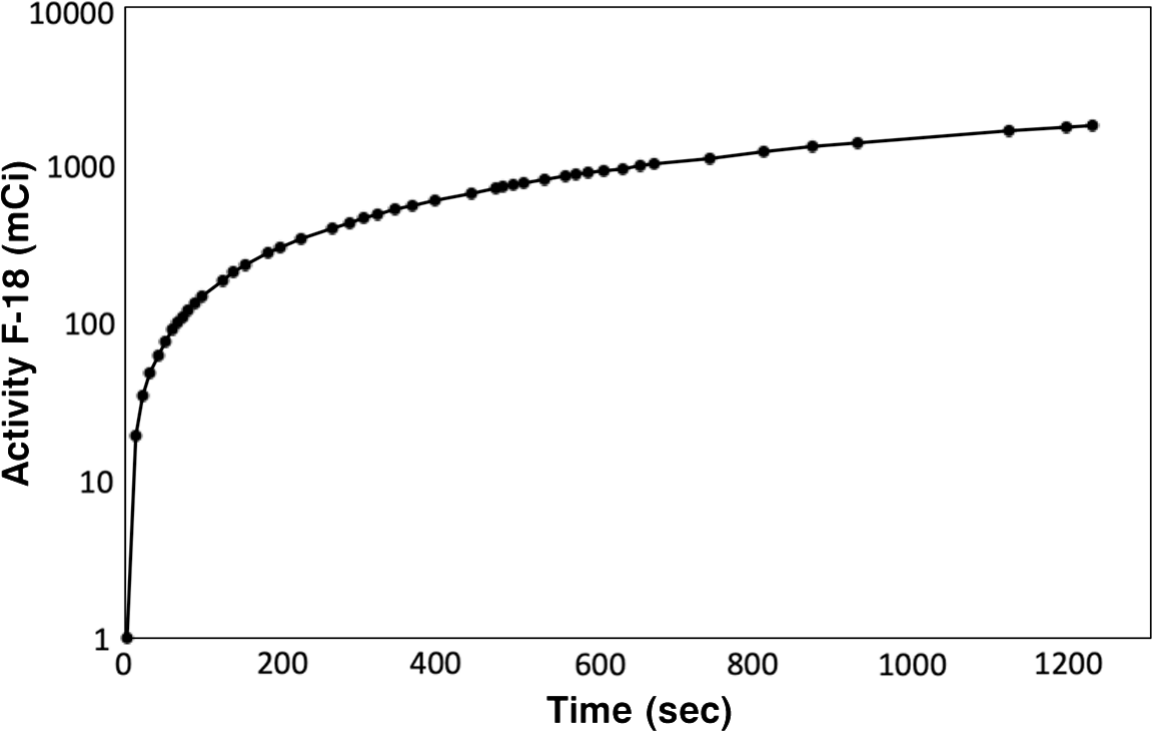
Activity (mCi) of ^18^F generated as a function of time (s) in an enriched ^18^O target using the cyclotron and experimental setup described previously with an operating current of 75 *μA*

Irradiations were performed on several Mo metal samples (Table 1) with various physical characteristics, i.e., mass, thickness, surface area, and volume, using the complementary neutrons produced during the production of ^18^F with a low-energy cyclotron. As previously discussed, the proton-induced transmutation of ^18^O to ^18^F results in the liberation of a free neutron, which is directly correlated to the yield of ^18^F produced in the ^18^O target. Shown in Fig. 3 is an ^18^F production curve for a 20-minute irradiation at 75 *μ*A yielding a total of ∼1.8 Ci of ^18^F at EOB. As every atom of ^18^F generated is equal to at least one neutron, then this would correspond to an average neutron flux of 5.2 × 10^11^ n/s with a total neutron output of $2.3 \times 10^{13}$ in the measured time period. With routine production schedules consisting of 4 to 5 h of operational beam time, the total average neutron output on a system equivalent to the one tested would range between 7.5 × 10^15^ to 9.4 × 10^15^ neutrons in a working day based solely on ^18^F transmutation, not considering other avenues of neutron production, for example, (*p*, *n*) reactions with the Havar window. In comparison to literature-reported values for neutron fluxes in a 16.5 MeV cyclotron, the value inferred from the ^18^F activity, i.e., 5.2 × 10^11^ n/s, is comparable to the one of Castillo [13], i.e., 6.0 × 10^11^ n/s, at 75 *μ*A, suggesting that the system is likely capable of outputting fluxes upwards of 1 × 10^12^ n/s with all other neutron producing reactions considered.

### Hybridised production of ^99m^Tc and ^101^Tc with complementary neutrons

Although Mo is characterised by seven naturally occurring isotopes, i.e., ^92^Mo (14.53%), ^94^Mo (9.16%), ^95^Mo (15.84%), ^96^Mo (16.67%), ^97^Mo (9.60%), ^98^Mo (24.39%), ^100^Mo (9.82%), only ^98^Mo and ^100^Mo provide direct routes to the appreciable formation of ^99^Mo when interacting with neutrons. For ^98^Mo, the fundamental interaction is via ^98^Mo(*n*, *γ*)^99^Mo. The neutron capture on ^98^Mo occurs across a wide range of neutron energies. The thermal neutron capture, i.e., 0.025 eV, on ^98^Mo has a cross-section (*σ*_thermal_) of approximately 0.137 b. In comparison to the fission-based production of ^99^Mo with thermalised neutrons, this value is nearly ${\sim} 270{\times}$ less for the equivalent irradiation using ^235^U as the fuel source. Furthermore, neutron capture reactions with thermal neutrons generally yield specific activities of hundreds of mCi/g at most depending upon the neutron flux, the amount of self-shielding effects, and the level of enrichment of ^98^Mo in the target. It is noted that enrichment of ^98^Mo can increase production yields up to 4× more than natural isotopic samples.

For higher energy neutrons, particularly in the resonance, i.e., 10-300 eV, and intermediate regions, i.e., 300 eV-0.05 MeV, ^98^Mo exhibits multitudes of enhanced resonance capture maxima. The resulting averaged cross-section of this region (*σ*_resonance_) approaches ∼7 b, which is more than 50× than that of *σ*_thermal_ for ^98^Mo, i.e., 0.130 b. Likewise, it has been established that the self-shielding of epithermal neutrons from other Mo isotopes is negligible comparative to interactions with ^98^Mo [19]. Because of the greater probability of interaction, it has been reported that specific activities up to ∼3.4 Ci/g and ∼15 Ci/g are achievable for natural and enriched targets, respectively, thus yielding significantly greater outputs in ^99^Mo [20]. The neutron capture behaviour of ^98^Mo is quite similar to ^100^Mo, where *σ*_thermal_ for ^100^Mo is 0.199 b and *σ*_resonance_ is 3.76 b.

The second accessible pathway for ^99^Mo production with neutrons via ^100^Mo(*n*,2*n*)^99^Mo only occurs for fast neutrons, (≥ 1 MeV) [21, 22]. The threshold reaction energy is approximately 8 MeV with a cross-section (*σ*_fast_) maximum of roughly 1.5 b befalling between 13 and 16 MeV. Relative to *σ*_thermal_ of ^98^Mo, this value is nearly 11× larger, although, for the averaged *σ*_resonance_ of ^98^Mo, it is about 5× less. However, because of the lower isotopic concentrations of ^100^Mo in comparison to ^98^Mo in natural samples, enriched materials can provide up to 10× the production. Due to the higher reaction threshold energy, this pathway is generally associated with production means where adequate fast neutron fluxes are present [23].

In order to determine the production of ^99^Mo/^99m^Tc via neutron activation in a Mo target, several Mo metal samples with varying geometries, i.e., cube versus sheet, were subjected to the neutron field within the cyclotron. Samples were placed in the vicinity of the highest neutron fluxes around the ^18^F target. Typically samples were arranged prior to ^18^F production for the given day and either measured at the end of the production day or after several days.

**Figure 4:**
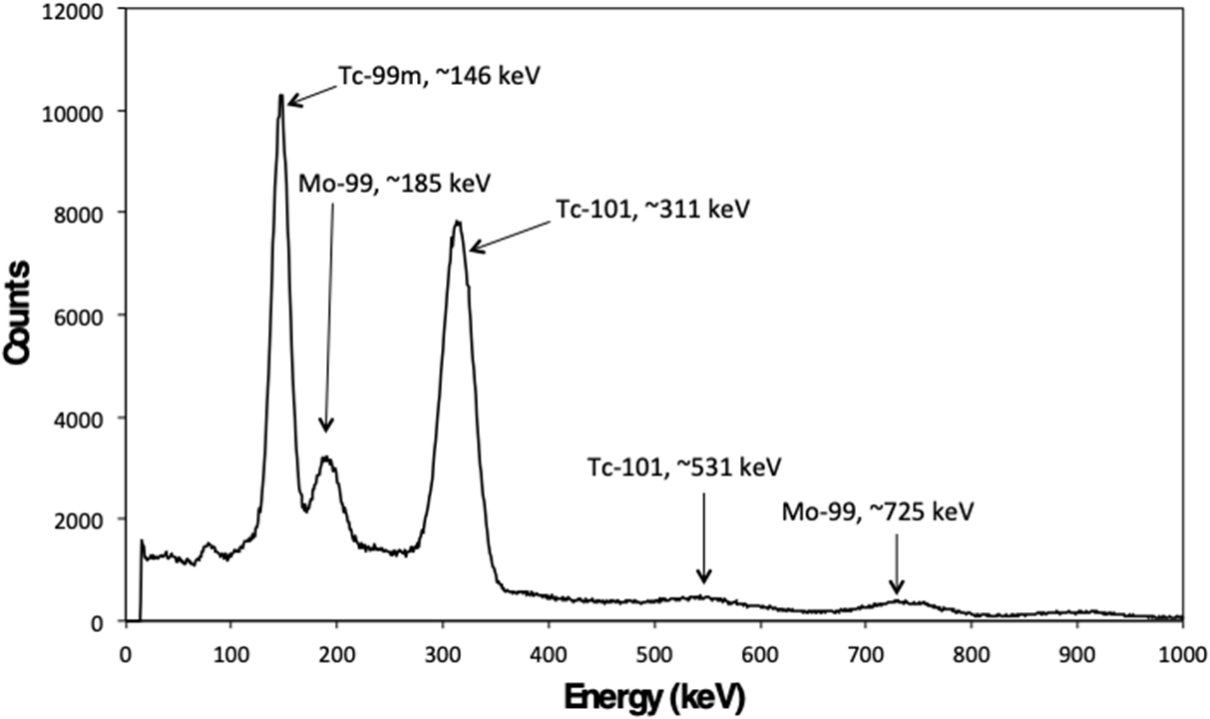
Gamma spectrum of Mo cube after ∼1.4 h post EOB showing characteristic peaks for ^99^Mo, ${}^{99 m}$Tc, and ^101^Tc after being irradiated in a neutron field for ∼4.6 h

Shown in Fig. 4 is the *γ*-ray spectrum of a Mo cube measured 1.4 h post-EOB following irradiation after 4.5 h in an un-moderated zone directly behind the ^18^F target, aligned with the incoming proton beam. The *γ*-ray spectrum shows the characteristic peaks for ^99^Mo at 185 keV and 725 keV, ^99m^Tc at 146 keV, and ^101^Tc at 311 keV and 531 keV. These are in good agreement with the reported gamma energies for ^99^Mo at 181.1 keV and 739.5 keV, ^99m^Tc at 140.5 keV, and ^101^Tc at 306.8 keV and 531 keV. The presence of ^99^Mo is most likely attributed to the neutron activation of ^98^Mo in the target, whereas ^99m^Tc present is a result of ^99^Mo decay.

The radioisotope ^101^Tc is also a daughter product, however, from ^101^Mo, formed via (*n*, *γ*) on ^100^Mo; the absence of any characteristic *γ*-ray peaks (E$\gamma = 229.1$ keV (2.20%), 257.1 keV (2.77%), 261.1 (20.6%), 359.1. keV (3.31%), 570.1 keV (6.62%)) from ^101^Mo is due to its shorter half-life (t$_{1/2} = 14.16$ min.). Unlike ^101^Mo, which is formed directly during the neutron irradiation, ^101^Tc being the daughter product still persists and can be seen as one of the more prominent peaks in the spectrum. Likewise, because the irradiation period (∼4.6 h) is sufficiently long compared to the half-life of ^101^Mo to achieve saturation, the concentration of ingrown ^101^Tc should be equivalent to that of ^101^Mo at EOB in the Mo target. Therefore, the total amount of ^101^Tc generated after EOB should be equal to the amount present at EOB plus the amount generated from residual ^101^Mo decay. The decay-corrected activity for the 311 keV peak of ^101^Tc in the sample was calculated to be ∼74 *μ*Ci at EOB.

**Figure 5:**
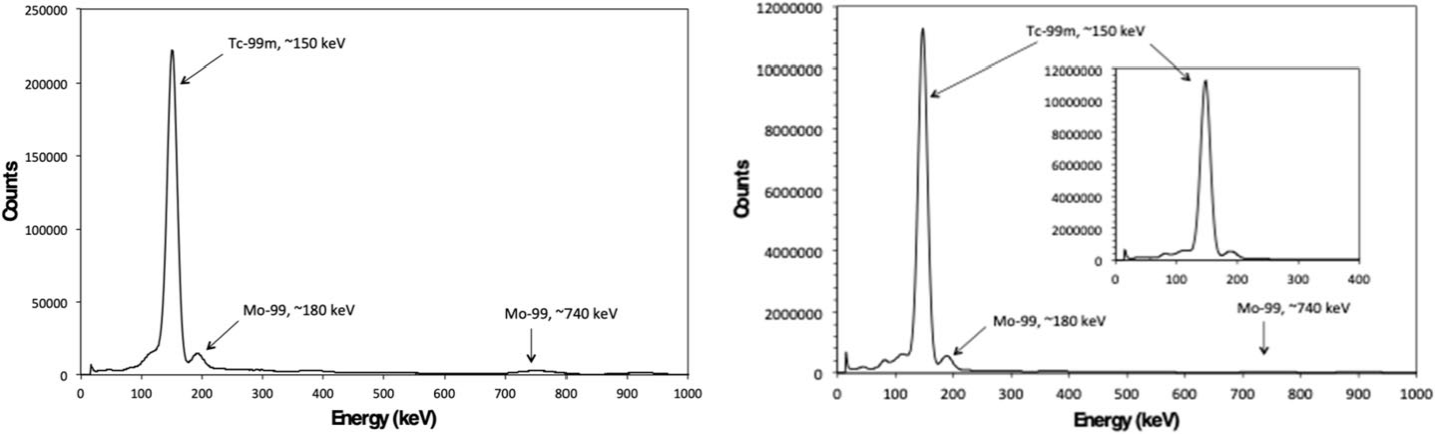
(Left) Gamma spectrum of Mo cube after 26 h post EOB showing characteristic peaks for ^99^Mo and ${}^{99 m}$Tc. (Right) Gamma spectrum (12 h count) of Mo foil acquired 24 h after EOB

Presented in Fig. 5 is the *γ*-ray measurement of the same Mo cube after 26 h post-EOB. The identified species in the spectrum were ^99^Mo with peaks at 180 keV and 740 keV, and ^99m^Tc with a peak at 150 keV. At this point, there was no remaining ^101^Tc. The peak-to-peak ratio of ^99m^Tc to ^99^Mo was determined to be 22.7, which is 6.6× greater than after 1.4 hr post EOB; this time post-EOB correlates to the near-maximum ingrowth of ^99m^Tc from ^99^Mo decay.

In Fig. 5, the *γ*-ray spectrum acquired 24 h after EOB of an irradiated Mo foil is presented. The foil was positioned underneath the ^18^F target and behind several layers of Styrofoam and irradiated within the neutron field for a total duration of 4.6 h. The *γ*-ray spectrum shows identifiable peaks indicative of ^99^Mo at 180 keV and 740 keV, and ^99m^Tc at 150 keV. As expected, no ^101^Mo and ^101^Tc were detectable at the time of/during measurement. The *γ*-ray spectrum here is also comparable with the one shown in Fig. 5 for the Mo cube.

The effect of various physical attributes, (i.e., volume, surface area) of the Mo targets and moderation / no moderation of the neutron field utilised in the production of ^99^Mo / ^99m^Tc were of particular interest. Three different samples, i.e., two Mo cubes (Cube 1 and Cube 2) and one Mo foil (Foil 1), were compared (Table 2), where all samples were irradiated for similar times (4.3 to 4.6 h) over the course of two production runs, one in which the samples were attached to the ^18^F used for production (∼2.7 h) and a second run during which they were located adjacently to the ^18^F target being operated (1.6 to 1.9 h); between the two production runs was a pause of approximately 1.5 h, however, for simplicity, ^99^Mo production only accounted for total irradiation time during operation. The effect of moderation was determined, where Cube 1 was placed in an un-moderated zone behind the ^18^F target, and Cube 2 was positioned underneath the target behind both a Styrofoam moderator and Foil 1. Final production activities and rates determined using Eq. (1) and Eq. (2), respectively, for ^99^Mo were normalised for decay after EOB and mass of the targets.

**Table 2:** Comparison of activities and reaction rates of ^99^Mo in various Mo metal samples after one production day of ^18^F in a low-energy cyclotron with neutrons generated through the ^18^O(*p*, *n*)^18^F reaction

	Cube 1	Cube 2	Foil 1
Mass	10.29075	10.2612	2.94533
Irradiation Time (s)	15540	16500	16500
Time after EOB (s)	98753	37736	39210
Count Time (s)	600	600	600
Total Counts ^99^Mo	391785	333068	197469
Total ^18^F Produced (Ci)	19.189	19.649	19.649
^99^Mo Activity (Bq)	1.28E+05	9.00E+04	5.16E+04
^99^Mo Activity (Ci)	3.45E–06	2.43E–06	1.40E–06
^99^Mo Specific Activity (Ci/g)	3.35E–07	2.37E–07	4.74E–07
Spec. Act. ^99^Mo (Ci/g)/^18^F (Ci)	1.75E–08	1.21E–08	2.41E–08
Ratio ^99^Mo (Ci)/^18^F (Ci)	1.80E–07	1.24E–07	7.10E–08
Reaction Rate of ^99^Mo (Bq)	2.87E+06	1.91E+06	1.10E+06
Reaction Rate of ^99^Mo (Ci)	7.77E–05	5.17E–05	3.93E–05
Reaction Rate of ^99^Mo (Ci/g)	7.55E–06	5.04E–06	1.34E–05

As shown in Table 2, the production and reaction rates of the two Mo cubes are presented. Both cubes exhibited identical volumes and surface areas, however, Cube 1 was subjected to no moderation of the incoming neutron field, whereas the neutron field was relatively moderated prior to interacting with Cube 2. The specific activities of ^99^Mo normalised to EOB and per gram of sample show that both samples yielded approximately 240 nCi/g to 340 nCi/g of ^99^Mo produced at EOB after one routine production day of ^18^F, or ∼12 nCi/g⋅Ci-^18^F to 18 nCi/g⋅Ci-^18^F. The reaction rates of formation of ^99^Mo in the samples were determined to be 5.0 *μ*Ci/g to 7.6 *μ*Ci/g, accounting for the irradiation time of each sample. From these values, it is seen that Cube 1 yielded slightly greater values of specific activity and reaction rates in comparison to Cube 2, although not significantly. When taking into consideration that the neutron flux generated perpendicular to the incoming proton beam is 70% of the beam generated in the same direction, the values of Cube 2 when adjusted for this are 343 nCi/g and 7.1 *μ*Ci/g for the specific activity and reaction rate, respectively, and are essentially identical for those of Cube 1. An explanation for this behaviour is as such: although Cube 2 experienced a lower neutron flux than that of Cube 1, the neutron energies due to moderation were more favourable for neutron capture reactions for Cube 2, thus allowing for equivalent production and reaction rates of ^99^Mo. As discussed previously, neutron capture on ^98^Mo is greatly favoured within neutron energies of 300 keV to 0.05 MeV, and it quickly diminishes with neutron energies outside of this region. Therefore, if the neutrons coming out of the target have energies greater than 0.05 MeV, which is likely the case, then there will be far fewer interaction probabilities for capture on ^98^Mo.

**Figure 6:**
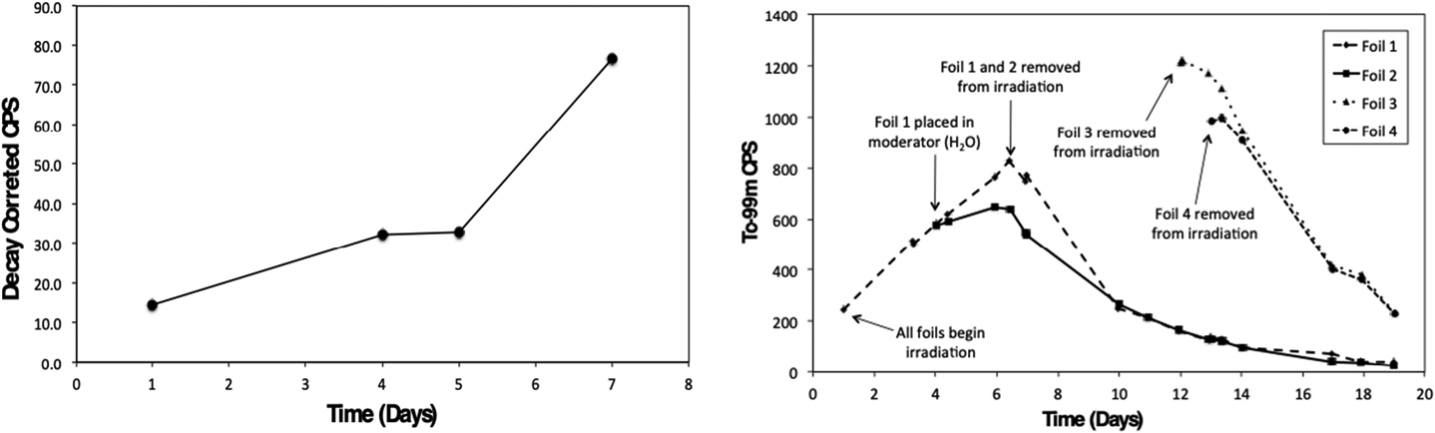
(Left) Production of ^99^Mo in a Mo foil tracked over several days of irradiation. Days 2 and 3 correspond to a weekend and no irradiations were performed. (Right) Presence of ${}^{99 m}$Tc (raw counts per second) in Mo foils 1–4 over the duration of the irradiation experiment

After establishing the approximate yield of ^99^Mo that could be generated in a single ^18^F production day, it was of interest to investigate and track production over the course of a several-day period. However, it is noted that because ^18^F production days were not all homogenous, where some days accounted for a single run and others multiple runs, attempting to extract any definitive behaviour of ^99^Mo / ^99m^Tc production was beyond the limit of the study and only broad trends in the data were to be considered. Figure 6 shows the relative activity of ^99^Mo produced over the course of several ^18^F production days. The starting day corresponds to a Friday, after which no irradiations were performed for the following two days over the weekend. Generally, the trend of the curve shows the relative build-in of ^99^Mo as a function of successive irradiations. Furthermore, a similar trend was also observed when tracking the ingrowth of ^99m^Tc in the samples, as shown in Fig. 6.

## Conclusion

The purpose of the study was to determine whether it was feasible to co-produce ^99m^Tc and ^101^Tc in parallel with ^18^F using a hybridised system based upon liberated neutrons from the ^18^O(*p*, *n*)^18^F and other potential associated (*p*, *n*) reactions generated with a low-energy biomedical cyclotron. From the data, it was demonstrated that the co-production of both isotopes under the tested conditions was feasible. It is noted that Mo sample design and placement were extremely rudimentary—if much higher quantities of ^99^Mo / ^99m^Tc or ^101^Mo / ^101^Tc were to be produced, possibilities for increasing yields, considering target mass/volume, irradiation time, and neutron flux could be applied for scaling. Linking a system like this with the appropriate separation platform for isolating Tc isotopes from irradiated low-specific activity (LSA) Mo targets, may become a distributed source of these and other medically relevant radioisotopes [3, 24]. Additionally, the possibility of leveraging at least some of the currently existing cyclotron infrastructures already in place could be seen as a huge advantage compared to building new reactors or facilities for radioisotope production.

## Data Availability

Data will be available from the authors upon reasonable request.
